# The impact of the COVID‐19 pandemic on the health of adults with intellectual impairment: evidence from two longitudinal UK surveys

**DOI:** 10.1111/jir.12866

**Published:** 2021-07-02

**Authors:** V. Totsika, E. Emerson, R. P. Hastings, C. Hatton

**Affiliations:** ^1^ Division of Psychiatry University College London London UK; ^2^ Centre for Educational Development Appraisal and Research University of Warwick Coventry UK; ^3^ Centre for Developmental Psychiatry and Psychology Monash University Melbourne Victoria Australia; ^4^ Centre for Disability Research, Faculty of Health and Medicine Lancaster University Lancaster UK; ^5^ Centre for Disability Research & Policy, Faculty of Health Sciences University of Sydney Sydney New South Wales Australia; ^6^ College of Nursing and Health Sciences Flinders University Adelaide South Australia Australia; ^7^ Department of Social Care and Social Work Manchester Metropolitan University Manchester UK

**Keywords:** COVID‐19 symptoms, health behaviours, intellectual impairment, mental health, physical health

## Abstract

**Background:**

People with an intellectual impairment experience high levels of social and health inequalities. We investigated the impact of COVID‐19 on the physical and mental health of people with intellectual impairment, controlling for demographic risk, socio‐economic circumstances and pre‐pandemic health levels.

**Method:**

Data were drawn from two UK birth cohorts that surveyed their participants on the impact of COVID‐19 in May 2020: the Millennium Cohort Study (20‐year‐old participants) and the British Cohort Survey (50‐year‐old participants). Health outcomes (COVID‐19 infection, COVID‐19 symptoms, self‐reported physical health, mental health, health service use and impact on health behaviours) were compared between people with and without intellectual impairment, adjusting for gender and ethnicity. Differences were further adjusted for self‐reported health pre‐pandemic and the impact of COVID‐19 on socio‐economic circumstances.

**Results:**

Controlling for gender and ethnicity, poor health was reported less often by younger adults [relative risks (RR): 0.44 95% confidence interval (CI) 0.23, 0.86] and more often by older adults (RR: 1.99 95% CI 1.45, 2.73) with intellectual impairment compared with peers. Older adults were also more likely to experience fever and loss of taste/smell. Adjusting for pre‐pandemic health and socio‐economic circumstances eliminated some differences in the older cohort, but not in the younger one.

**Conclusion:**

In young adulthood, the impact of COVID‐19 on health outcomes was not negative. The pattern was reversed in later adulthood, although differences were mostly eliminated after adjustment suggesting a socio‐economic and age gradient of COVID‐19 impacts on intellectual impairment.

## Introduction

In the United Kingdom, the first UK‐wide COVID‐19 lockdown began on 26 March 2020 with restrictions remaining in place until mid‐June. Initial reports hypothesised that the impact of COVID‐19 and associated lockdown restrictions would be severe for people with intellectual disability (ID) in terms of infection risk and mental health (Courtenay and Perera 2020). Emerging evidence suggests that people with an ID receiving statutory services experienced more severe impact on their health than the rest of the population (Hüls *et al*. 2020; Landes *et al*. 2021; Turk *et al*. 2020). The aim of the present study is to investigate the impact of COVID‐19 on the wider group of people with an intellectual impairment, a hidden majority that is known to experience multiple disadvantage in terms of socio‐economic inequalities, physical and mental health, and life chances (Holland *et al*. 2002; Søndenaa *et al*. 2008; Peltopuro *et al*. 2014).

Intellectual impairment is present in about 15% of the UK population (McManus *et al*. 2018). About 13% are individuals with cognitive limitations, sometimes defined by a low IQ (IQ 70–85) and often referred to as having borderline intellectual functioning (Salvador‐Carulla *et al*. 2018; Martínez‐Leal *et al*. 2020). About 2% to 2.5% of the population has an ID (IQ < 70 and significant limitations in adaptive skills) (Maulik *et al*. 2011; Salvador‐Carulla *et al*. 2018). We collectively refer to the two groups as having an intellectual impairment. People with more severe levels of ID will be known to services but most people with a mild ID or borderline intellectual functioning are not identified as such by statutory health and social care services in the United Kingdom. They live in the community independently or with family, and they may sometimes be supported by voluntary or charitable organisations.

Evidence from studies prior to COVID‐19 indicated that people with intellectual impairment experience higher levels of socio‐economic deprivation, poorer physical and mental health outcomes, more loneliness and fewer social relationships (Peltopuro *et al*. 2014; McManus *et al*. 2018; Hassiotis *et al*. 2019) compared with the rest of the population. People with intellectual impairment may be at greater risk during a pandemic due to the nature of their impairment (cognitive and/or adaptive skill limitations), the well‐established social inequities they experience (poverty and deprivation) and the greater prevalence of health problems. As yet, we have virtually no evidence about the impact of the COVID‐19 pandemic on this multiply‐disadvantaged group. Part of the difficulty in researching this lies in the identification of this population: in the absence of statutory or clinical identification, population‐representative national studies with cognitive assessments offer the best route to identification.

The aim of our study is to investigate the impact of COVID‐19 on the health of people with an intellectual impairment in two UK birth cohort studies, including participants at the age of 20 and 50 years. We investigate whether health inequities due to COVID‐19 are present in relation to physical health status, mental health, COVID‐19 infection, health service use, and health behaviours, and if so, whether this is attributable to the presence of intellectual impairment, over and above the effect of demographic risk, socio‐economic circumstances and pre‐pandemic health levels.

## Method

Data for the present study were drawn from the Millennium Cohort Study (MCS) and the British Cohort Study (BCS70), two UK birth cohorts. MCS is the most recent one, established in 2000 to follow up a population‐representative sample of 19 286 children born in the United Kingdom (Joshi and Fitzsimons 2016). MCS waves followed up participants at the ages of 3, 5, 7, 11, 14 and 17 years, and in 2020, a COVID‐19 survey was sent to those who participated at the 17‐year follow up. The BCS70 birth cohort study followed up 17 198 UK children born during 1 week in 1970. BCS70 data were collected shortly after birth and at ages 5, 10, 16, 26, 30, 34, 38, 42 and 46 years (Elliott and Shepherd 2006). In 2020, a COVID‐19 survey was sent to those who participated in the 46‐year follow up. The COVID‐19 survey was developed by the Centre for Longitudinal Studies and the MRC Unit of Lifelong Health and Ageing and sent to all UK national surveys managed by Centre for Longitudinal Studies. The intention was to explore the impact of the COVID‐19 pandemic and associated lockdown on the lives of participants during May 2020, in the first lockdown period.

### Participants

In MCS, 2645 cohort members participated in the COVID‐19 survey (26.6% response rate). Of those, 2609 were the first cohort member. The remaining 36 participants were twins or triplet siblings of the first cohort member. The first cohort member had been randomly selected at study entry in 2000. Given the very small number of siblings, we focused our analyses on 2609 participants who were the first cohort child to be selected for participation in MCS. Of those, we were able to classify 2588 cohort members with regard to intellectual impairment as defined in measures. Two‐hundred and sixteen participants were identified with intellectual impairment (II), representing 8.35% of the unweighted, and 12.08% of the weighted sample (*N*_IIw_ = 311).

MCS participants were aged between 19 and 20 years old at the time of data collection (May 2020). Overall, 49% of respondents were male (55.5% men in the II group; 47.9% in the non‐II group). Most were of White ethnicity (88.0% in the non‐II group; 71.7% in the II group). Over half of the participants were in education at the start of the pandemic (56.0% in the non‐II group; 54.8% in the II group). The vast majority (over 85% in both groups) lived with their parents.

In BCS, 4132 cohort members participated in the COVID‐19 survey (40.4% response rate). Of these, we were able to classify 3987 cohort members (96.4%) with regard to intellectual impairment as defined in measures. Two‐hundred and fifty six participants were identified with intellectual impairment (II), representing 6.4% of the unweighted and 12.8% of the weighted sample (*N*_IIw_ = 465).

BCS participants were aged 50 years old at the time of data collection (May 2020). Overall, 50.5% of respondents were male, and almost all respondents were of White ethnicity (97.0%). Three quarters (74.5%) were in paid employment or self‐employed.

#### Measures

##### Health and infection

Participants self‐reported on their physical health status in a 5‐point item (‘In general, would you say your health is …’) in the COVID‐19 wave and the previous data wave (age 17 and 46 in MCS and BCS70, respectively). Responses of fair and poor health were collapsed into one response category, and responses of excellent, very good and good were collapsed into a second response category. Mental health was measured using the K6 (Kessler *et al*. 2002) in MCS and an abbreviated version of the Malaise Inventory in BCS70 (Rodgers *et al*. 1999), two well‐established measures of psychiatric morbidity in community populations. Recommended cut‐points (Kessler *et al*. 2003; Brown and Peters 2019) were used in the present study to indicate poor mental health. Participants reported whether they had been infected with COVID‐19 (yes confirmed by test, yes strongly suspected, no, and unsure) and whether they had experienced three key COVID‐19 symptoms [National Health Service (NHS) 2020]: cough (dry or mucous), loss of taste or smell, and fever. As COVID‐19 testing was extremely limited in the United Kingdom during the study period, data on the experience of key COVID‐19 symptoms are considered a proxy of likely COVID‐19 infection.

##### Health service use

Participants indicated whether they had been tested for Coronavirus, whether they had been contacted by health officials as at risk of severe illness because of underlying medical problems (‘shielding’) and whether they had surgery or medical appointments cancelled since the outbreak of COVID‐19.

##### Impact on health behaviours

Participants were asked to indicate the amount of exercise, fruit and vegetable consumption, sleep, alcohol, smoking and vaping they did in the month before the Coronavirus outbreak and since the start of the outbreak. We estimated the difference in health behaviours between these two time points, and whether there was an overall deterioration in all health behaviours.

##### Intellectual impairment

Intellectual impairment was identified in each birth cohort by scores lower than one standard deviation below the mean of a general cognitive factor that was derived from standardised cognitive assessments measured during childhood (ages 5 and 10 in BCS70; Hassiotis *et al*. 2019 and ages 3, 5 and 7 years in MCS; Totsika *et al*. 2020). In total, 3006 MCS participants were identified with intellectual impairment (*n* = 17 of those were identified through parent reports of special educational needs and teacher reports of significant academic underachievement in reading, writing and maths at age 7), representing 13.7% (weighted prevalence) of 19 244 MCS first cohort participants, and 2543 BCS70 participants representing 14.7% of 17 198 BCS70 participants. Under participants, we reported how many people with intellectual impairment from MCS and BCS70 participated in the COVID‐19 of data collection.

#### Approach to analysis

Comparisons of health outcomes (self‐reported physical health, mental health, COVID‐19 infection and symptoms, health service use and impact on health behaviours) between adults with and without intellectual impairment were performed first unadjusted and then adjusted for gender and ethnicity (white vs. ethnic minority). Any outcomes that were found to still differ significantly between the two groups were adjusted further for self‐reported health at the previous wave of data collection (pre‐COVID), and the impact of COVID‐19 on socio‐economic circumstances (managing financially in the 3 months prior to COVID‐19, change in financial security, food insecurity, and use of foodbank during lockdown). Analyses were conducted separately in MCS and BCS70 and were weighted to account for the survey's sampling design (MCS) and attrition (MCS and BCS70). We report prevalence rate ratios [i.e. relative risks (RR)] derived from log‐binomial models and Poisson models with robust standard errors (Knol *et al*. 2012).

## Results

Table 1 presents descriptive statistics on health outcomes for people with intellectual impairment and those without at age 20 (MCS) and age 50 years (BCS70). In MCS, very few differences were seen between the two groups (Table 2); most notably young adults with intellectual impairment were less likely to report their health was poor compared with their peers without impairment (RR:0.44 [95% confidence interval (CI): 0.23, 0.86], before and after adjustment for gender and ethnicity). Older adults with intellectual impairment (BCS70) were more likely to report poorer health (RR: 1.99 [95% CI: 1.45, 2.73) and experience one or more of the three key COVID symptoms (RR: 1.47 [95% CI: 1.06, 2.04]), even after adjusting for gender and ethnicity (Table 2).

**Table 1:** Health outcomes in people with intellectual impairment (II) and those without

Variable	MCS: age 20 years		BCS70: age 50 years	
	II	Non‐II	II	Non‐II
	*N* (%)	*N* (%)	*N* (%)	*N* (%)
Self‐reported health				
Fair/poor health	15 (4.8)	244 (9.5)	144 (31.0)	470 (14.8)
Poor mental health	37 (16.0)	393 (19.5)	81 (19.8)	576 (19.6)
COVID‐19 infection				
Yes	12 (3.8)	129 (5.7)	22 (4.7)	317 (10.0)
Unsure	74 (23.7)	479 (21.1)	145 (31.2)	740 (23.4)
No	22 (72.6)	1657 (73.2)	298 (64.1)	2112 (61.6)
COVID‐19 key symptoms				
Fever	14 (4.4)	57 (2.5)	41 (8.9)	96 (3.1)
Cough (dry or mucous)	62 (20.0)	449 (20.0)	108 (23.5)	558 (17.8)
Loss of taste or smell	6 (2.1)	61 (2.7)	38 (8.3)	104 (3.3)
One or more key symptoms	64 (20.5)	505 (22.5)	139 (30.3)	627 (20.0)
Health service use				
Tested for COVID‐19	11 (3.6)	49 (2.2)	10 (2.2)	112 (3.6)
Asked to shield	3 (1.0)	56 (2.5)	30 (6.5)	184 (5.8)
Had surgery or medical appointment cancelled	36 (11.5)	23.5 (10.4)	84 (18.1)	378 (11.9)
Impact on health behaviours				
Smoking more	18 (5.6)	117 (5.1)	9 (9.9)	81 (14.9)
Vaping more	13 (4.1)	77 (3.4)	14 (24.6)	74 (27.2)
Drinking more	26 (8.3)	121 (5.3)	41 (14.0)	245 (10.3)
Exercising less	99 (31.9)	639 (28.2)	107 (27.4)	893 (30.4)
Less fruit & veg	26 (8.4)	327 (14.5)	87 (21.9)	469 (16.3)
Sleeping less	79 (25.3)	489 (21.6)	59 (14.6)	825 (27.9)

BCS70, British Cohort Study; MCS, Millennium Cohort Study.

**Table 2:** The association of intellectual impairment with health outcomes

Variable	MCS: age 20 years		BCS70: age 50 years	
	Unadjusted relative risk	Adjusted relative risk^a^	Unadjusted relative risk	Adjusted relative risk^a^
Self‐reported health status				
Fair/poor health	**0.44 (0.23, 0.84)**	**0.44 (0.23, 0.86)**	**2.08 (1.46–2.96)**	**1.99 (1.45–2.73)**
Poor mental health	0.82 (0.52, 1.30)	0.88 (0.54, 1.43)	1.01 (0.66–1.53)	1.02 (0.68–1.52)
COVID‐19 infection				
Infection likely (yes and unsure)	1.02 (0.73, 1.43)	1.08 (0.77, 1.52)	1.08 (0.82–1.42)	1.06 (0.82–1.38)
Key COVID‐19 symptoms				
Fever	1.78 (0.67, 4.63)	1.59 (0.58, 1.52)	**2.92 (1.11–7.69)**	**2.78 (1.09–7.03)**
Cough	1.00 (0.64, 1.56)	1.01 (0.64, 1.61)	1.32 (0.89–1.96)	1.26 (0.87–1.83)
Loss of taste or smell	0.76 (0.25, 2.51)	0.77 (0.25, 2.40)	**2.49 (1.08–5.77)**	**2.47 (1.18–5.16)**
One or more key symptoms	0.91 (0.59, 1.41)	0.93 (0.59, 1.45)	**1.51 (1.08–2.12)**	**1.47 (1.06–2.04)**
Health service use				
Tested for COVID‐19	1.69 (0.39, 7.30)	1.81 (0.37, 8.78)	0.61 (0.27–1.34)	0.52 (0.22–1.26)
Asked to shield	0.42 (0.12, 1.45)	0.37 (0.11, 1.32)	1.12 (0.59–2.12)	1.12 (0.60–2.10)
Had surgery or medical appointment cancelled	1.11 (0.63, 1.97)	1.26 (0.71, 2.42)	1.52 (0.97–2.37)	1.56 (1.00–2.44)
Impact on health behaviours				
Deterioration in health behaviours	1.07 (0.82, 1.39)	1.07 (0.82, 1.39)	0.90 (0.71–1.14)	0.90 (0.72–1.13)
Smoking more	1.10 (0.41, 2.95)	1.26 (0.46, 3.44)	0.66 (0.27–1.59)	0.90 (0.37–2.19)
Vaping more	1.20 (0.28, 5.15)	1.36 (0.32, 5.73)	0.91 (0.34–2.42)	0.83 (0.32–2.15)
Drinking more	1.55 (0.67, 3.61)	1.80 (0.78, 4.16)	1.36 (0.74–2.49)	1.39 (0.76–2.56)
Less exercise	1.13 (0.82, 1.55)	1.10 (0.80, 1.52)	0.90 (0.64–1.28)	0.93 (0.67–1.28)
Less fruit & veg	**0.58 (0.36, 0.96)**	**0.57 (0.35, 0.93)**	1.35 (0.87–2.09)	1.32 (0.86–2.04)
Less sleep	1.17 (0.80, 1.72)	1.14 (0.75, 1.71)	**0.52 (0.37–0.74)**	**0.51 (0.36–0.73)**

BCS70, British Cohort Study; MCS, Millennium Cohort Study.

^a^ Relative risk derived from log‐binomial or Poisson models with robust standard errors (Knol *et al*. 2012). adjusted for gender and ethnicity.

Health outcomes that were significantly different after accounting for the effect of gender and ethnicity were modelled further. At this next step, we adjusted for self‐reported health pre‐pandemic: at age 17 MCS participants with intellectual impairment were as likely to report poor health as their peers (RR: 0.76, 95% CI: 0.37, 1.56), whereas at age 46 BCS70, participants with intellectual impairment were significantly more likely to report poor health compared with peers (RR: 1.74, 95% CI: 1.26, 2.41). Controlling for self‐reported health at the previous wave attenuated most of the associations (Table 3), with two exceptions: self‐reported health among MCS participants with intellectual impairment was still significantly better than that of their peers, and one COVID‐19 symptom (loss of taste or smell) was still significantly more likely among BCS70 participants with intellectual impairment. The final step adjusted for current socio‐economic circumstances: struggling to manage financially in the 3 months prior to COVID‐19, negative change in financial security, food insecurity, and use of foodbank. Intellectual impairment was now associated with a higher risk of experiencing fever and loss of taste or smell in BCS70 participants who were 50 years old, and a lower risk of reporting poor/fair health in MCS participants who were 20 years old.

**Table 3:** Adjusting relative risk associated with intellectual impairment for health at the previous wave and current socio‐economic circumstances

Step: Adjusted for …	Fever (BCS70)	Loss of taste/smell (BCS70)	Presence of one or more key symptoms (BCS70)	Current self‐reported health being fair/poor (BCS70)	Current self‐reported health being fair/poor (MCS)
+ previous wave self‐reported health	2.21 (0.87–5.64)	**2.19 (1.02–4.68)**	1.35 (0.98–1.85)	1.33 (0.95–1.86)	**0.42 (0.22, 0.83)**
+ managing financially in the 3 months prior to COVID, change in financial security, food insecurity and use of foodbank	**3.01 (1.60–5.65)**	**2.76 (1.41–5.40)**	1.31 (0.95–1.80)	1.19 (0.89–1.58)	**0.34 (0.15, 0.74)**

BCS70, British Cohort Study; MCS, Millennium Cohort Study.

## Discussion

The impact of the first COVID‐19 lockdown in the United Kingdom on the health of people with intellectual impairment was restricted for an early adulthood cohort (age 20 years) but more pronounced in an age 50 years cohort.

Young adults with intellectual impairment were no more likely than their peers without impairment to be infected by COVID‐19, experience key COVID‐19 symptoms, or a deterioration of their health behaviours. Mental health problems in May 2020 were at similar levels between these two groups. Health service use was also largely similar between young adults with intellectual impairment and their peers without impairment. At the age of 50, people with intellectual impairment were more likely than their peers to experience fever and loss of smell or taste, or report one or more key COVID‐19 symptoms.

At the age of 20, people with intellectual impairment were more likely to self‐report better health compared with peers, even after controlling for gender, ethnicity, self‐reported health status at 17 years, and current socio‐economic circumstances. This finding is consistent with recent findings of better health outcomes among people in California, USA, who receive services for intellectual or developmental disabilities and reside in the community compared with those who do not have intellectual or developmental disabilities (Landes *et al.* 2021).

The Landes *et al*. (2021) study did not control for the effect of age, but our findings indicated that the effect on health may be reversed by age 50, when people with intellectual impairment are more likely to report poorer health and key COVID‐19 symptoms. These health effects persisted after controlling for gender and ethnicity, although some were no longer present after controlling for pre‐pandemic health status, and current socio‐economic circumstances. Taken together, the pattern of findings in the present study suggests a largely socio‐economic and age gradient of COVID‐19 impacts on health for individuals with intellectual impairment. The socio‐economic and age gradient of COVID‐19 impacts on health is consistent with evidence over the same time period from the overall population (Davies *et al*. 2020) and also those with ID (Henderson *et al*. 2021). The present findings highlight the increased vulnerability of older adults with an intellectual impairment.

Evidence is accruing that the impact of COVID‐19 on the health of the population is unevenly distributed across socio‐economic strata [Office of National Statistics (ONS) 2020; Chen and Krieger 2021]. Health inequities are expected to increase as the impact of COVID‐19 on social inequalities increases further due to rise in unemployment and loss of earnings (Parkes and McNeil 2020; Tinson 2020). During the first lockdown when the present data were collected, the United Kingdom had not yet experienced the steep rise in unemployment seen in subsequent months, while loss of earnings was largely mitigated by financial measures put in place across the United Kingdom. In this study, the impact of COVID‐19 on the socio‐economic circumstances of people with intellectual impairment was more pronounced in the older cohort, where 50 years olds with intellectual impairment were more likely to report financial insecurity and use of foodbank. It is very likely that social inequities may have increased since the first lockdown because of the steep rise in unemployment and the subsequent variation (in time and geographical location) of financial support offered since. It is thus likely that health inequities between those with intellectual impairment and those without will become more pronounced as social and economic inequities increase into 2021.

The findings need to be considered in light of the study's limitations. Most notably, data were drawn from two COVID‐19 data waves with very high levels of attrition from the main cohort studies. Potential sampling bias associated with high attrition indicates caution when generalising the proposed health impacts to all adults with intellectual impairment in the United Kingdom. As both surveys recruited participants from the community, people with intellectual impairment in residential services were not included: these are likely to be people with more severe ID. Those in residential care and those at the more severe end of the ID spectrum were more likely to experience adverse health outcomes from COVID‐19 (Perera *et al*. 2020; Public Health England 2020; Henderson *et al*. 2021). It is thus likely that findings in the present study are an underestimate of the population effect.

Overall, findings from the present study suggest that during the first lockdown period, older adults with intellectual impairment were more likely to experience an adverse impact on their health due to COVID‐19, and this increased vulnerability was largely associated with the higher level of socio‐economic and health inequities they were experiencing before COVID‐19. Future studies need to examine health inequities in intellectual impairment during subsequent periods of the COVID‐19 pandemic when the economic impacts of the pandemic were more pronounced.

## Source of Funding

No external funding supported the research presented in the current paper.

## Conflict of Interest

The authors report no conflict of interest.

## Data Availability

The data that support the findings of this study are available in the UK Data Archive at https://www.data‐archive.ac.uk/, reference UKDA Study Number 8658.
